# Ephrin-A1-Mediated Dopaminergic Neurogenesis and Angiogenesis in a Rat Model of Parkinson's Disease

**DOI:** 10.1371/journal.pone.0032019

**Published:** 2012-02-20

**Authors:** Xuefeng Jing, Hideto Miwa, Takahiro Sawada, Ichiro Nakanishi, Tomoyoshi Kondo, Masayasu Miyajima, Kazushige Sakaguchi

**Affiliations:** 1 Department of Molecular Cell Biology and Molecular Medicine, Institute of Advanced Medicine, Wakayama Medical University, Wakayama, Japan; 2 Department of Neurology, Wakayama Medical University, Wakayama, Japan; 3 Laboratory Animal Center, Wakayama Medical University, Wakayama, Japan; National Institutes of Health, United States of America

## Abstract

Cells of the neural stem cell lineage in the adult subventricular zone (SVZ) respond to brain insult by increasing their numbers and migrating through the rostral migratory stream. However, in most areas of the brain other than the SVZ and the subgranular zone of the dentate gyrus, such a regenerative response is extremely weak. Even these two neurogenic regions do not show extensive regenerative responses to repair tissue damage, suggesting the presence of an intrinsic inhibitory microenvironment (niche) for stem cells. In the present study, we assessed the effects of injection of clustered ephrin-A1-Fc into the lateral ventricle of rats with unilateral nigrostriatal dopamine depletion. Ephrin-A1-Fc clustered by anti-IgG(Fc) antibody was injected stereotaxically into the ipsilateral lateral ventricle of rats with unilateral nigrostriatal lesions induced by 6-hydroxydopamine, and histologic analysis and behavioral tests were performed. Clustered ephrin-A1-Fc transformed the subventricular niche, increasing bromodeoxyuridine-positive cells in the subventricular area, and the cells then migrated to the striatum and differentiated to dopaminergic neurons and astrocytes. In addition, clustered ephrin-A1-Fc enhanced angiogenesis in the striatum on the injected side. Along with histologic improvements, behavioral derangement improved dramatically. These findings indicate that the subventricular niche possesses a mechanism for regulating both stem cell and angiogenic responses via an EphA–mediated signal. We conclude that activation of EphA receptor–mediated signaling by clustered ephrin-A1-Fc from within the lateral ventricle could potentially be utilized in the treatment of neurodegenerative diseases such as Parkinson's disease.

## Introduction

Self-renewal and differentiation of somatic stem cells are regulated by the stem cell environment (niche) via as yet undefined mechanisms [1]. Glial fibrillary acidic protein (GFAP)–positive cells give rise to neurons and interact directly with other cells in the subventricular zone (SVZ) and subgranular zone (SGZ). They are, therefore, regarded as neural stem cells [2], [3]. Ependymal cells surrounding the lateral ventricle are in close proximity with these cells in the SVZ and produce stem cell–activating factors [4], [5]. Blood vessel endothelial cells are another critical component of the SVZ [6]; dividing neural stem/progenitor cells (NSPCs) are tightly apposed to SVZ capillary vessels [7]. These findings suggest that GFAP–positive cells, ependymal cells, and capillary endothelial cells may function as niche cells.

Lesions to the brain initiate the proliferation of NSPCs and neurogenesis in the SVZ [8], [9]. A major proportion of proliferating neuroblasts in the SVZ migrate to the olfactory bulb and become interneurons [10]. Because the striatum is closely associated with the SVZ, NSPCs there would be an ideal source of cellular replacements for the damaged striatum. In rats with lesions of the nigrostriatal pathway, the natural response of cellular proliferation in the SVZ is weak and disappears within a few weeks [9]. In this case, some NSPCs originating in the SVZ can migrate to the adjacent striatum, but few differentiate to neurons [9], [10].

Ephrins signal via EphA and EphB receptor tyrosine kinases (forward signaling), and Eph receptors also transmit signals via ephrins (reverse signaling) [11]. EphAs bind to ephrin-As anchored to the cell membrane via a glycosylphosphatidylinositol linkage. EphBs bind to ephrin-Bs, which have a transmembrane domain and a short cytoplasmic domain. Eph/ephrin signals play important stimulatory and inhibitory roles in boundary formation, cell migration, repulsive axon guidance [12], and regulation of neuronal growth cone development [13]. They also regulate cell-matrix interactions [14], [15], [16] and cell proliferation [17], [18]. Recent reports suggest that Eph receptors regulate angiogenesis in embryonic and adult tissues [19].

Neurogenesis is regulated by many factors, among which several ephrins and their Eph receptors play important roles. They are differentially expressed on distinct cell types of the neurogenic niche, and also have differential functions on stem cell proliferation, survival and differentiation. EphA2, EphA3 and EphA4 receptors are expressed in the SVZ, and involved in NSPC differentiation towards neuronal lineage in vitro [20]. EphA4 appears to be expressed in the adult neurogenic niches exclusively by neural stem cells, and function to maintain neural stem cell proliferation [21]. Complimentary expression of ephrin-A2 in transit-amplifying cells and neuroblasts and that of EphA7 on ependymal cells and neural stem cells (NSCs) inhibits neural progenitor proliferation through reverse signaling [22]. EphA7 is shown to be involved in the modulation of apoptosis of neural progenitors during embryonic development [23]. Infusion of the ectodomain of either EphB2 or ephrin-B2 into the lateral ventricle disrupts migration of neuroblasts and increase cell proliferation [24]. Ephrins B2 and B3 and their receptor EphB1 suppress proliferation and survival of NSPCs and migration of neuroblasts in the SVZ, rostral migratory stream (RMS) and SGZ [25], [26]. EphB2 induces proliferation of SVZ cells in vitro [27]. EphB3 signal suppresses NSPC proliferation in a p53-dependent manner [28]. Ephrin-B1 is shown to be critical for maintenance of NSPCs [29].

Fibroblast growth factor receptors (FGFRs) are also tyrosine kinases that attract and phosphorylate a variety of signaling proteins when activated, forming an assembly of signaling complexes that activates multiple signaling pathways [30]. FGF promotes proliferation of neural stem cells [31], [32], and FGF activity has been implicated in the maintenance of stem cell niches in vivo [33]. We recently reported that interaction of activated EphA4 with FGFRs augments proliferative signaling via fibroblast growth factor receptor substrate 2α (FRS2α) [34], [35] and that coactivated FGFRs stimulate migration-related signal through RhoA by phosphorylating Ephexin1 [36]. These findings suggested to us that there might be an intrinsic regulatory mechanism of ephrin-A/EphA–mediated signaling in the neural stem cell niche. In the present study, we show that ephrin-A/EphA–mediated signaling plays a role in neuronal regeneration in a rat model of Parkinson's disease.

## Materials and Methods

### Ethics Statement

All animal experiments were performed in compliance with the guidelines of the Animal Experiment Committee of the Wakayama Medical University in accordance with the Act on Welfare and Management of Animals (Law No. 105, Japan), the Fundamental Guidelines for Proper Conduct of Animal Experiment and Related Activities in Academic Research Institutions (Japanese Ministry of Education, Culture, Sports, Science and Technology, Notice No. 71, 2006), and the Standards Relating to the Care and Management of Laboratory Animals and Relief of Pain (Japanese Ministry of Environment, Notice No. 88, 2006). Our proposed studies entitled, “Studies on the effect of subventricular stem cell stimulation in situ in a rat model of Parkinson's disease”, have been approved by the committee under the institutional approval numbers of #141 (from April 1, 2004 to March 31, 2006) and #258 (from June 1, 2006 to May 31, 2011).

### Preparation of Clustered Ephrin-A1-Fc, Clustered IgG(Fc), and FGF2

Recombinant mouse ephrin-A1 fused by means of a polypeptide linker to the Fc portion of human IgG_1_ (ephrin-A1-Fc) was purchased from Sigma-Aldrich (St. Louis, MO; Cat. #E9902). To prepare clustered ephrin-A1-Fc, 20 µg ephrin-A1-Fc was incubated with 48 µg rabbit anti-human IgG(Fc) antibody (Jackson ImmunoResearch Laboratories, West Grove, PA; Cat. #309-005-008) in 30 µl phosphate-buffered saline (PBS) at 4°C for ≥1 hour. As a control, a human IgG(Fc) fragment (Jackson ImmunoResearch Laboratories; Cat. #009-000-008) was subjected to the same treatment. Recombinant human FGF2 was from R&D Systems (Minneapolis, MN) and dissolved in PBS containing 0.1% heat-inactivated bovine serum albumin (BSA) at 10 µg/ml for storage and diluted further with PBS for injection or infusion. For injection, 3 µg ephrin-A1-Fc or IgG(Fc) or 100 ng FGF2 was used in a volume of 3–4 µl. For infusion through a 100 µl microosmotic pump, 2–3 µg/day ephrin-A1-Fc or IgG(Fc) or 50 ng/day FGF2 was infused into the lateral ventricle. Ephrin-A1-Fc and IgG(Fc) were used with or without clustering as specified in each study.

### Unilateral Lesioning of the Nigrostriatal Dopaminergic Pathway

Male Sprague Dawley rats (7–9 weeks old) were injected stereotaxically with 2 µl saline containing 6 µg/µl 6-hydroxydompamine (6OHDA) and 0.2% ascorbic acid into the right medial forebrain bundle (coordinates: anterior/posterior −2.2 mm, lateral [right] 1.5 mm relative to bregma, dorsoventral −8.0 mm from the dural surface) under anesthesia. Rats were examined for typical rotational behavior after intraperitoneal apomorphine injection (0.5 mg/kg) 6–8 weeks after injection of 6OHDA. Rats that rotated on average more than 100 times in 20 minutes when measured in 3 successive 20-minute periods were regarded as having severe dopamine deficiency (unilaterally lesioned rats) [37]. These unilaterally lesioned rats were always used 6–8 weeks after lesioning with 6OHDA.

### Intraventricular Injection

Rats were anesthetized with ketamine (60 mg/kg) and xylazine (10 mg/kg), and reagents were injected or were infused with a microosmotic pump (Alzet 1007D; Durect Corp, Cupertino, CA) into the lesioned side (usually right side) of the lateral ventricle (coordinates: anterior/posterior −0.9 mm, lateral [right] 1.5 mm relative to bregma, dorsoventral −5.0 mm from the dural surface). Reagents included clustered IgG(Fc) (3 µg/injection or 3 µg infusion/day), unclustered ephrin-A1-Fc (3 µg/injection or 3 µg infusion/day), clustered ephrin-A1-Fc (3 µg/injection or 3 µg infusion/day), or FGF2 (100 ng/injection or 50 ng infusion/day). In some experiments, rats were infused with these reagents for 1 week after injection of 1 µg Chloromethylbenzamido-DiI (CM-DiI, C-7001, Molecular Probes) dissolved in 2 µl of solution containing 5% DMSO in 300 mM sucrose. Rats were examined for rotational behavior periodically after intraperitoneal injection of apomorphine (0.5 mg/kg).

### RNA Extraction and RT-PCR

Tissue facing the ventricle (∼100 µm in depth) was microdissected under a stereomicroscope. RNA extraction was performed with TRI reagent (Sigma-Aldrich). Reverse transcription (RT) was performed with MuLV reverse transcriptase and an oligo d(T)_16_ reverse transcription primer in 20 µl (Gene Amp RNA PCR kit; Roche, Indianapolis, IN). Polymerase chain reaction (PCR) was performed with 35 2-step cycles of 30 seconds at 94°C and 45 seconds at 58°C using 3 µl of the RT product and GoTaq polymerase (Promega, Madison, WI). The PCR products were fractionated on 2% agarose gels, and bands were stained with ethidium bromide and detected under ultraviolet irradiation. Amplified PCR products were confirmed to have the expected base sequence by DNA sequencing from both ends. Primers for reverse transcription polymerase chain reaction (RT-PCR) are listed in Table S1.

### Immunohistochemistry

Rats were anesthetized and perfused with ice-cold PBS followed by 4% paraformaldehyde, and brains were removed and fixed in 4% paraformaldehyde overnight at 4°C. Brains were cryoprotected in a PBS series containing increasing concentrations (10%, 20%, 30%) of sucrose. Indirect immunohistochemistry was performed with 40-µm-thick, free-floating, coronal sections. For detection of bromodeoxyuridine (BrdU), sections were rinsed in PBS and incubated in formamide in 2× saline-sodium citrate (SSC) (50%, v/v) for 2 hours at 65°C followed by a 15-minute rinse in 2× SSC at 37°C. The DNA was then treated with 2 N HCl for 30 minutes at 37°C followed by a 10-minute rinse in 0.1 M boric acid buffer, pH 8.5. Then, the following steps were performed as routine immunohistochemical staining. Tissue sections were permeabilized with 0.25% Triton X-100 in PBS mixed with 1% normal rabbit serum and incubated with primary antibodies at 4°C for 48 hours. After a wash in PBS, they were incubated with second antibodies (DAKO) conjugated to horseradish peroxidase (for 3,3′-diaminobenzidine staining) or Alexa Fluor 488 and Alexa Fluor 568 (Molecular Probes) at 4°C for 2 hours, followed by a wash in PBS. Sections were counterstained with 4′,6-diamino-2-phenylindole (DAPI; Molecular Probes) or with Wheat Germ Agglutinin-Alexa Fluor 488 Conjugate (Molecular Probes) as necessary, and mounted with Gel/Mount (Biomeda Corp, Foster City, CA). Staining with 3,3′-diaminobenzidine was detected with a Nikon Eclipse E800 microscope or a Keyence BZ-9000 microscope. Immunofluorescence was detected with a Keyence BZ-9000 microscope, Nikon Eclipse E800 fluorescence microscope, Nikon Eclipse TE300 inverted microscope, BioRad Radiance 2000 confocal microscope, or a Zeiss LSM5 Pascal confocal microscope. Antibodies used in this study were listed in Table S2.

When quantification is needed, we counted the number or measured the area of the marker-labeled cells in 8 areas (9×10^4^ µm^2^/area and 10 µm thickness) per animal from 4 equally spaced coronal sections of striatum or sagittal sections of the granule cell layer of the olfactory bulb, and the calculated average value was taken as representing the animal. The selected areas in the striatum were 500–800 µm lateral from the lateral wall of the lateral ventricle and around the center between the dorsal and ventral edge of the striatum in each section.

### Immunoprecipitation and immunoblotting

Immunoblotting of Ephs was performed with brain tissue lysate that was taken from the brain region (100 µm thickness) surrounding the ventricles, and lysate containing 200–300 µg total protein was incubated with unclustered ephrin-A1-Fc or specific EphA antibodies followed by immunoprecipitation with protein A agarose. Lysis buffer contained 50 mM HEPES, 1% Triton X-100, 5 mM ethylenediaminetetraacetic acid, 50 mM NaCl, 10 mM sodium pyrophosphate, 50 mM sodium fluoride, 1 mM sodium orthovanadate, and protease inhibitors (1 mM phenylmethylsulfonyl fluoride, 1 µM aprotinin, 1 µM leupeptin, and 1 µM pepstatin A). Immunoprecipitated proteins were fractionated by sodium dodecyl sulfate-polyacrylamide gel electrophoresis, blotted onto polyvinylidene fluoride membranes (Millipore, Billerica, MA), and incubated with EphA-specific antibodies (Santa Cruz Biotechnology, Santa Cruz, CA) or mouse monoclonal antiphosphotyrosine antibody (clone 4G10; Millipore, Billerica, MA). Immunodetection was performed with an Immobilon Western Blotting Detection System (Millipore, Billerica, MA). Antibodies used in this study were listed in Table S2.

### BrdU Labeling

BrdU (20 mg/ml) dissolved in PBS was injected into the peritoneal cavity (80 mg/kg) at intervals of several hours, as indicated.

### Stereologic Cell Counting

Stereologic analysis was performed under blinded conditions on coded slides. For each rat, we analyzed the entire striatum on the lesioned and reagent (ephrin-A1-Fc or IgG[Fc])-infused side. Numbers of BrdU(+) cells were determined in every tenth section in a series of 40 µm coronal sections throughout the rostrocaudal extent of the striatum with a semiautomatic stereology system (Stereoinvestigator; MicroBrightField, Williston, VT) and a 5× objective to trace the striatum. Volume was determined by summing traced areas of each brain and multiplying by intersection distance (400 µm). Our criterion for selecting individual BrdU(+) nuclei was the presence within the counting frame or touching the right or top frame lines but not touching the left or bottom lines.

### Statistical Analysis

All the values were expressed as mean ± SD. Comparison of 2 values was performed with Student *t*-test, or with a nonparametric analysis, Mann Whitney test. Comparison of ≥3 values was performed by one-way analysis of variance followed by Tukey multiple comparison test.

## Results

### Expression of EphA Receptors, FGFRs and their downstream molecules in Adult Subventricular Tissue and Activation of EphA4 by Clustered Ephrin-A1-Fc

**Figure 1:**
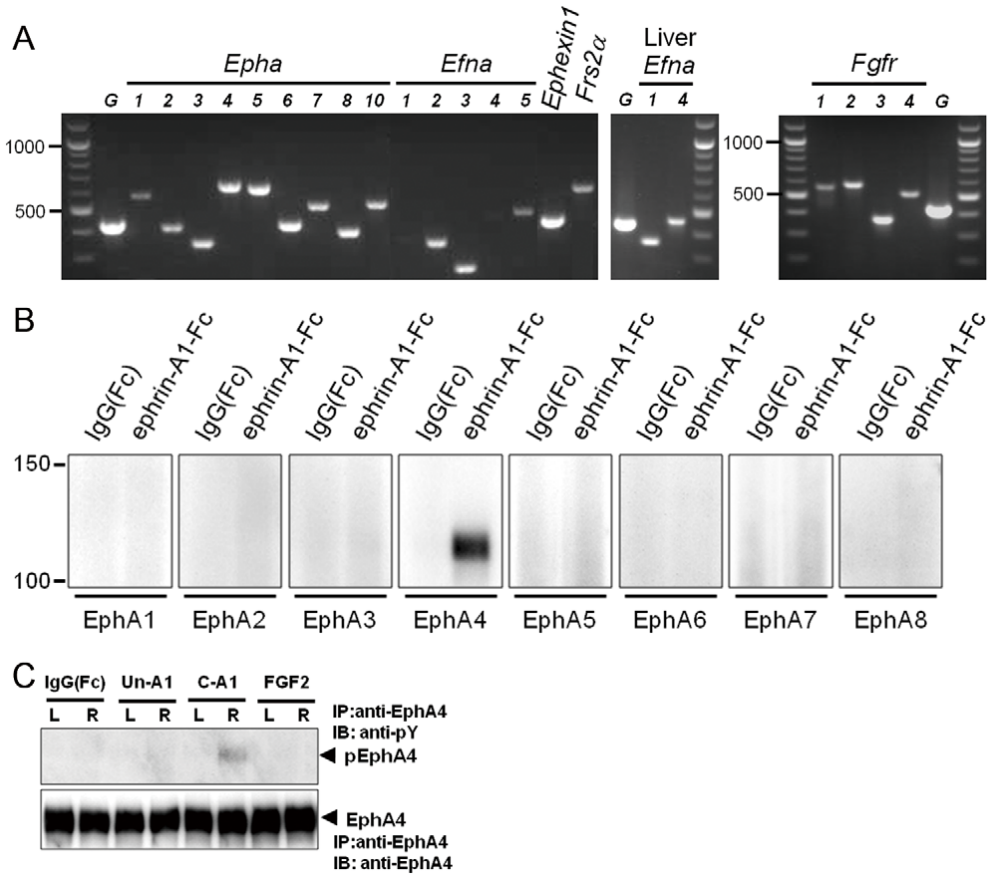
Expression of EphA–related signaling molecules in subventricular zone cells. (**A**) RT-PCR for *Epha*s, *Efna*s, *Fgfr*s, *Ephexin1*, and *Frs2α*. *G*: Glyceraldehyde 6-phosphate dehydrogenase. For *Efna1* and *Efna4*, liver RNA was also used as control. (**B**) Detection of EphA receptors that binds to ephrin-A1-Fc in rat brain lysate. Rat brain subventricular cell lysate was incubated with ephrin-A1-Fc, precipitated by protein A-agarose, and immunoblotted with the antibodies for EphAs. (**C**) EphA4 phosphorylation in tissue surrounding the lateral ventricle. In rats with lesioned unilaterally in the nigrostriatal dopaminergic pathway, subventricular tissue was collected 18 hours after single injection of clustered IgG(Fc) (3 µg), unclustered ephrin-A1-Fc (Un-A1) (3 µg), clustered ephrin-A1-Fc (C-A1) (3 µg), or FGF2 (100 ng). The tissue lysate (450 µg protein) was immunoprecipitated with anti-EphA4 antibody followed by immunoblotting with anti-phosphotyrosine (pY) antibody and anti-EphA4 antibody.

To examine expression of the molecules related to the ephrin/Eph signal transduction pathway, we extracted RNA from the microdissected subventricular area (100 µm thickness from the surface of the ventricles), and performed RT-PCR using the primers shown in Table S1 (Fig. 1A). All *Epha* mRNAs and those for ephrin-A2, -A3, and -A5 (*Efna2*, *Efna3*, and *Efna5*) were expressed in the SVZ, but those for ephrin-A1 and -A4 (*Efna1 and Efna4*) were barely detectable or undetectable. mRNAs for *Fgfrs, Frs2α, and Ephexin1* were also expressed.

To investigate which EphA receptors possibly bind to the recombinant ephrin-A1-Fc, we incubated the tissue lysate from the subventricular area with unclustered ephrin-A1-Fc, followed by immunoprecipitation with protein A-agarose. Only EphA4 was co-immunoprecipitated and detectable (Fig. 1B). These findings suggest that the clustered ephrin-A1-Fc used in the current study predominantly binds to EphA4 in the SVZ. We obtained the same results using the microdissected subventricular tissue from normal rats and rats with unilaterally lesioned nigrostriatal dopaminergic pathway described below. Studies were repeated 3 times in both normal and lesioned rats, and showed the same results.

To examine the effect of ephrin-A1-Fc on the cells of SVZ, we used rats with a unilaterally lesioned nigrostriatal dopaminergic pathway because the evaluation of striatal dopamine depletion and repletion is standardized. The nigrostriatal dopaminergic pathway of rats was lesioned 6–8 weeks before intraventricular injection of clustered ephrin-A1-Fc or other reagents. Clustered or unclustered ephrin-A1-Fc, clustered IgG(Fc), or FGF2 was injected stereotaxically into the lateral ventricle on the lesioned side. Immunoblotting with anti-phosphtyrosine antibody 18 hours after injection showed that clustered ephrin-A1-Fc phosphorylated EphA4, however, FGF2, unclustered ephrin-A1-Fc or clustered IgG(Fc) did not (Fig. 1C). The effect was limited to the lesioned side where clustered ephrin-A1-Fc was injected.

### Effect of Clustered Ephrin-A1-Fc in Rats with Unilateral Nigrostriatal Pathway Lesions

**Figure 2:**
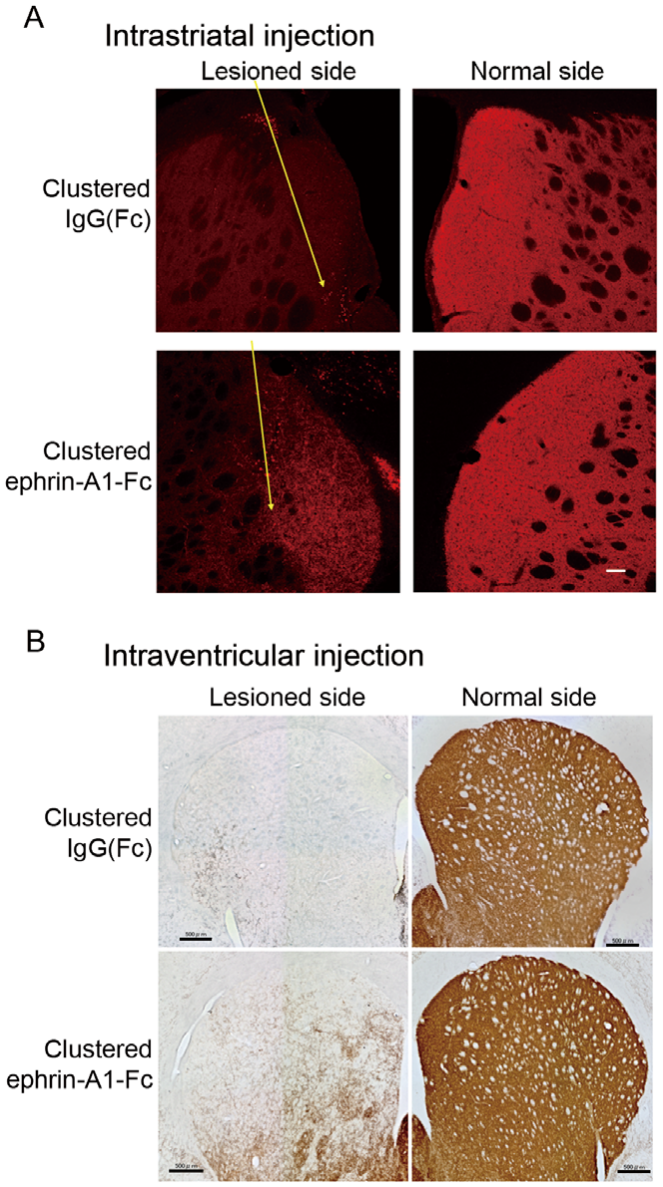
Effect of clustered ephrin-A1-Fc injection in rats with chemical lesions in the unilateral nigrostriatal dopaminergic pathway. (**A**) Effect of intrastriatal injection of clustered ephrin-A1-Fc on the regeneration of dopaminergic cells. Rats with unilateral nigrostriatal lesions were injected with clustered IgG(Fc) (upper panels) or ephrin-A1-Fc (lower panels) into the lesioned side of the striatum near the subventricular zone. Rats were killed 6 weeks after injection. Brains were sectioned coronally, and sections were stained immunohistochemically for tyrosine hydroxylase (TH). Yellow arrows indicate the course of needles used for injection of clustered IgG(Fc) or ephrin-A1-Fc. Scale bar: 100 µm. (**B**) Entire striatal coronal view of TH–positive cells. Clustered IgG(Fc) or ephrin-A1-Fc was injected into the ventricle ipsilateral to the lesioned side, and coronal sections of the entire striatum were stained for TH. Upper panels, clustered IgG(Fc)-injected rat; lower panels, clustered ephrin-A1-Fc-injected rat. Scale bar: 500 µm.

To roughly locate the area of brain that responds to ephrin-A1-Fc treatment, we injected clustered ephrin-A1-Fc into the striatum close to the SVZ. Injection of clustered ephrin-A1-Fc (3 µg) induced the appearance of tyrosine hydroxylase (TH)–positive (+) neuronal fibers on the side of the ventricle 4–12 weeks after injection, but not on the striatal side (Fig. 2A). Intraventricular injection of clustered ephrin-A1-Fc ipsilateral to the lesion increased the number of TH(+) neurons on the lesioned side of the striatum. These were clearly localized as multiple islands in the ephrin-A1-Fc-injected, lesioned side of the striatum 4–12 weeks after injection, although they were not yet distributed as densely as on the normal side (Fig. 2B; Fig S1). We confirmed similar intrastriatal and intraventricular effects of clustered ephrin-A1-Fc in 2–3 rats at each time point after injection (4, 6, 8, and 12 weeks).

### Behavior of Rats with Unilateral Dopamine Depletion before and after Treatment with Clustered Ephrin-A1-Fc

**Figure 3:**
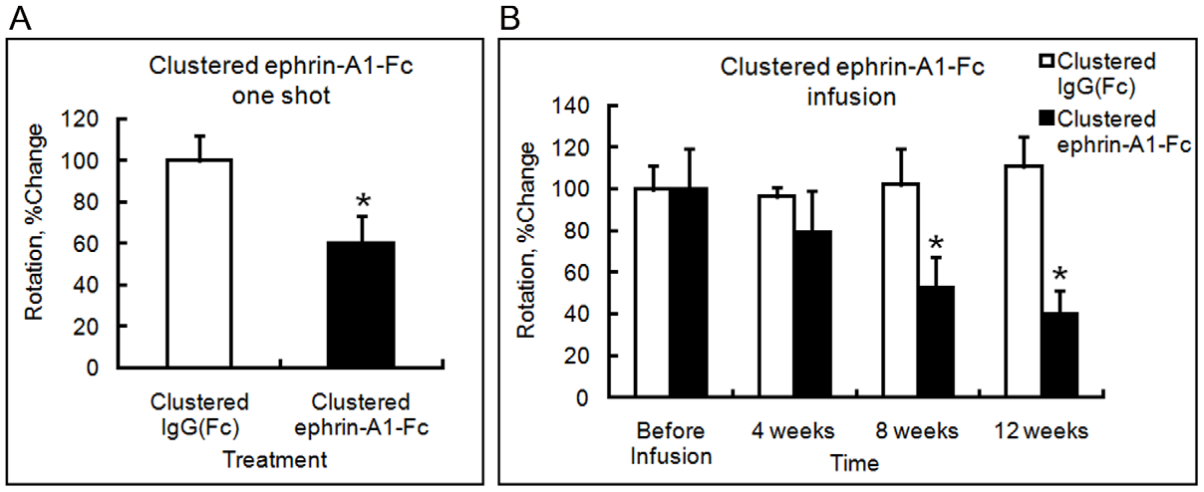
Behavioral effects of clustered ephrin-A1-Fc injection into the lateral ventricle. Effect of a single injection (3 µg) (**A**) or a 1-week infusion with a micro-osmotic pump (**B**) of clustered ephrin-A1-Fc (2 µg/day) or control (clustered IgG[Fc]). Production of rats with unilateral nigrostriatal lesions and evaluation of behavior after intraperitoneal injection of apomorphine were performed as described in the Materials and Methods. (**A**) Behavior was analyzed just before and 6 weeks after injection of clustered IgG(Fc) or ephrin-A1-Fc. In each animal, the value of rotation frequency at 6-week point was adjusted to that before injection. Mean of the adjusted values in control animals at 6-week point was set at 100%. Error bars represent SD. *p<0.01 (n = 7). (**B**) Behavioral analysis was performed in lesioned rats infused with clustered IgG(Fc) (n = 5; white bars) or clustered ephrin-A1-Fc (n = 5; black bars) every 4 weeks up to 12 weeks from the start of infusion. In each group, the mean rotation value before infusion was taken as 100%. Error bars represent SD. *p<0.01 compared to the control.

Rotation frequency after injection of clustered IgG(Fc) or ephrin-A1-Fc was analyzed before and 6 weeks after treatment in the rats treated with a single injection, and before and 4–12 weeks after treatment in the rats treated with a 7-day infusion. A single intraventricular injection of clustered ephrin-A1-Fc (3 µg) significantly decreased rotation frequency to about 60% of that of IgG(Fc) injection at 6 weeks after treatment, (*p*<0.01, n = 7) (Fig. 3A). A 7-day continuous ventricular infusion of clustered ephrin-A1-Fc (2 µg/day) also decreased rotation frequency at 8 and 12 week points, to 53% and 40%, respectively, as compared to the value before infusion (*p*<0.01, n = 5) (Fig. 3B).

### Distribution of BrdU(+) Cells from the Ventricular Side to the Striatum after Intraventricular Injection of Clustered Ephrin-A1-Fc

**Figure 4:**
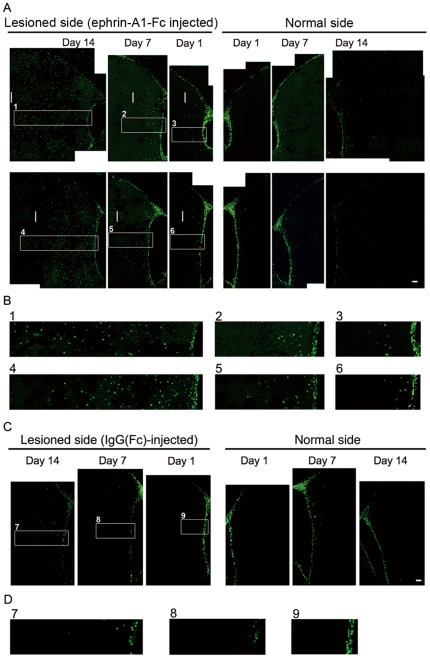
Distribution of BrdU(+) cells in the striatum after intraventricular injection of clustered ephrin-A1-Fc. (**A**) Pulse-chase experiments of BrdU labeling of proliferating cells. Unilaterally lesioned rats were injected with a single dose of clustered ephrin-A1-Fc (3 µg) into the lateral ventricle of the lesioned side, followed by 3 intraperitoneal injections of BrdU at 6-hour intervals. Coronal sections of brains were examined 1, 7, and 14 days after injection of clustered ephrin-A1-Fc. BrdU staining is shown as fine green dots in the striatum. TH staining was used to detect the lesioned side of the brain (not shown). Lesioned sides are shown on the left, and normal sides are shown on the right. The numbers of days after clustered ephrin-A1-Fc injection (Day 0) are shown above the panels. Upper panels represent the anterior part of the striatum, and lower panels represent the middle region of the striatum. Vertical white bars show the intra-striatal distribution front of BrdU(+) cells. (**B**) Enlarged photomicrographs of the numbered regions in (A). (**C**) The same as (A) except that clustered IgG(Fc) (3 µg) instead of clustered ephrin-A1-Fc was injected as control. Only the middle region of the striatum is shown. (**D**) Enlarged photomicrographs of the numbered regions of (C). Scale bar: 100 µm. These are the typical results of 3 separate experiments for each treatment.

To study possible distribution of BrdU(+) cells along the SVZ-striatum axis, rats were treated with a single intraventricular injection of clustered ephrin-A1-Fc (3 µg) to the lesioned side of the lateral ventricle, followed by 3 intraperitoneal injections of BrdU (80 mg/kg) at 6-hour intervals, and killed 1, 7, or 14 days after the ephrin injection. Coronal brain sections were stained for nuclear BrdU incorporation. Cells on both sides of the SVZ showed extensive BrdU incorporation 1 day after ephrin injection (6 h after the last BrdU injection). The BrdU(+) cells ipsilateral to the ephrin injection side, which is also the lesioned side, showed dense BrdU(+) cell populations moving toward the periphery of the lesioned striatum from the SVZ over 14 days (moving front shown by vertical lines), whereas BrdU(+) cells did not migrate to the striatum on the nonlesioned and non-ephrin-injected sides (Fig. 4A, B). Unilaterally lesioned rats injected with clustered IgG(Fc) followed by intraperitoneal BrdU injection did not show such effect (Fig. 4C, D). These studies were repeated in 3 rats for each treatment and each time point, and showed similar results. A possible diffusion of ephrin-A1-Fc into the striatum was also examined immunohistochemically in the brain sections using fluorescence-labeled anti-human IgG(Fc) antibody. The results showed no detectable staining (data not shown).

### Tracking of Migrating BrdU(+) Cells with CM-DiI from the Subventricular Zone to the Striatum and Olfactory Bulb

**Figure 5:**
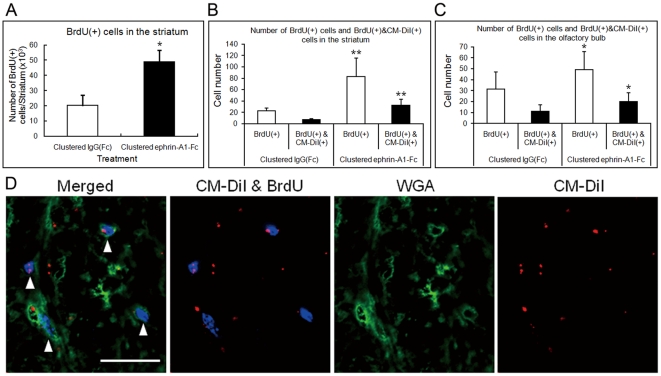
Tracking BrdU(+) cells following intraventricular infusion of clustered ephrin-A1-Fc. Unilaterally lesioned rats were infused with clustered IgG(Fc) or clustered ephrin-A1-Fc into the lateral ventricle of the lesioned side for 1 week with simultaneous intraperitoneal injection of BrdU. (**A**) Number of BrdU(+) cells in the striatum counted with a stereologic counting system. n = 4 for the clustered IgG(Fc)-infused and ephrin-A1-Fc-infused groups. Error bars represent SD. *p<0.05 (Mann Whitney test, n = 4) compared to clustered IgG(Fc). (**B**) Tracking the cells labeled with BrdU and CM-DiI in the striatum. Brain slices were stained for BrdU and with Wheat Germ Agglutinin (WGA), and confocal 3D micrographs were taken at 1-µm intervals. Then, ten serial confocal micrographs were compiled for one all-in-focus micrograph, and the number of cells labeled with BrdU or co-labeled with both BrdU and CM-DiI was counted in defined areas as described in the Materials and Methods. **p<0.01 (n = 8) compared to IgG(Fc). (**C**) Tracking of the cells labeled with BrdU and CM-DiI in the olfactory bulb. Study protocols are the same as in (B). The labeled cells in the granule cell layer of the olfactory bulb were counted. *p<0.05 (n = 8) compared to IgG(Fc). (**D**) A typical confocal micrograph showing the relative localization of BrdU (blue), WGA (green) and CM-DiI (red) in a cell level in the striatum. Scale bar, 20 µm; arrowheads, cells labeled with both BrdU and CM-DiI.

To study whether clustered ephrin-A1-Fc increased the number of the BrdU(+) cells in the striatum, and to compare the effect with that of unclustered ephrin-A1-Fc or FGF2, the unilaterally lesioned rats were infused with clustered IgG(Fc) (3 µg/day), unclustered ephrin-A1-Fc (3 µg/day), clustered ephrin-A1-Fc (3 µg/day), or FGF2 (50 ng/day) into the lateral ventricle of the lesioned side continuously for 1 week with simultaneous intraperitoneal injection of BrdU (80 mg/kg) twice a day (every 12 hours). Brains were removed 6 weeks after the start of infusion, sliced into 40-µm thick coronal sections, and stained for BrdU. We counted the number of BrdU(+) cells in 8 areas (9×10^4^ µm^2^) per animal in the striatum and in the granule cell layer of the olfactory bulb as described in the Materials and Methods section (Fig S2). In the striatum, clustered ephrin-A1-Fc, but not unclustered ephrin-A1-Fc or FGF2, increased the number of BrdU(+) cells by ∼4-fold over clustered IgG(Fc) treatment. In the olfactory bulb, the normal target of subventricular neuroblasts migration [38], clustered ephrin-A1-Fc and FGF2, but not unclustered ephrin-A1-Fc, increased the number of BrdU(+) cells significantly over clustered IgG(Fc) treatment. To clarify if the increase of BrdU(+) cells was extended to all over the striatum, we performed stereologic counting to quantify precisely the number of BrdU(+) cells throughout the striatum on the treated side after infusion of clustered ephrin-A1-Fc or IgG(Fc) and found that clustered ephrin-A1-Fc increased the number significantly (∼2.5-fold) over IgG(Fc) treatment (Fig. 5A).

Then, to study whether the ephrin-A1-Fc-induced distribution of BrdU(+) cells in the striatum was caused by migration of NSPCs from the SVZ, we injected CM-DiI into the lesioned side of the lateral ventricle right before infusion of clustered ephrin-A1-Fc and covalently labeled the cells exposed to the ventricular surface with the fluorescent dye. Brains were removed 6 weeks after the start of ephrin-A1-Fc infusion, sliced into 40-µm thick coronal sections, and stained for BrdU. Brain slices were stained for BrdU and with Wheat Germ Agglutinin conjugated with Alexa Fluor 488 (Molecular Probes), and confocal 3D micrographs were taken at 1-µm intervals. Then, ten serial confocal micrographs were compiled for one all-in-focus micrograph, and the number of cells labeled with BrdU or co-labeled with both BrdU and CM-DiI was counted in selected areas. When cells 500–800 µm lateral from the ventricular surface were examined 6 weeks after the start of clustered ephrin-A1-Fc infusion, the ephrin infusion enhanced almost 4 times the number of double-labeled cells over infusion of clustered IgG(Fc) (Fig. 5B). Unclustered ephrin-A1-Fc did not increase the number of double-labeled cells beyond that induced by IgG(Fc) (data not shown). In the granule layer of the olfactory bulb, we also found that the number of double-labeled cells significantly increased after treatment with clustered ephrin-A1-Fc (Fig. 5C). However, the ratio of BrdU(+)CM-DiI(+) cells over BrdU(+) cells, which is around 1/3, stayed almost same in treatment with clustered IgG(FC) or ephrin-A1-Fc in both striatum and olfactory bulb. The relative localization of CM-DiI and BrdU in a cell level was clearly shown by staining with labeled Wheat Germ Agglutinin that binds to cell surface carbohydrates (Fig. 5D). The results indicate that many BrdU(+) cells were labeled with CM-DiI at their surface.

These findings clearly show that substantial percentage of BrdU(+) cells had evidence of exposure to the lateral ventricle and migrated from the ventricular area into the striatum. On the other hand, there is no clear evidence on the origin of non-CM-DiI-labeled BrdU(+) cells. Several reasons can be considered for this non-labeling with CM-DiI; technical difficulty in detecting the CM-DiI label, NSPCs not exposed to the ventricle, mitogenically active cells residing in the striatum, etc. However, the finding that clustered ephrin-A1-Fc increases the numbers of both BrdU(+)CM-DiI(+) and BrdU(+) cells in the striatum as well as in the olfactory bulb without changing their ratio suggests that the double labeled cells are representing the BrdU(+) cells moving from the SVZ.

### Transformation of Subventricular Niche Cells

**Figure 6:**
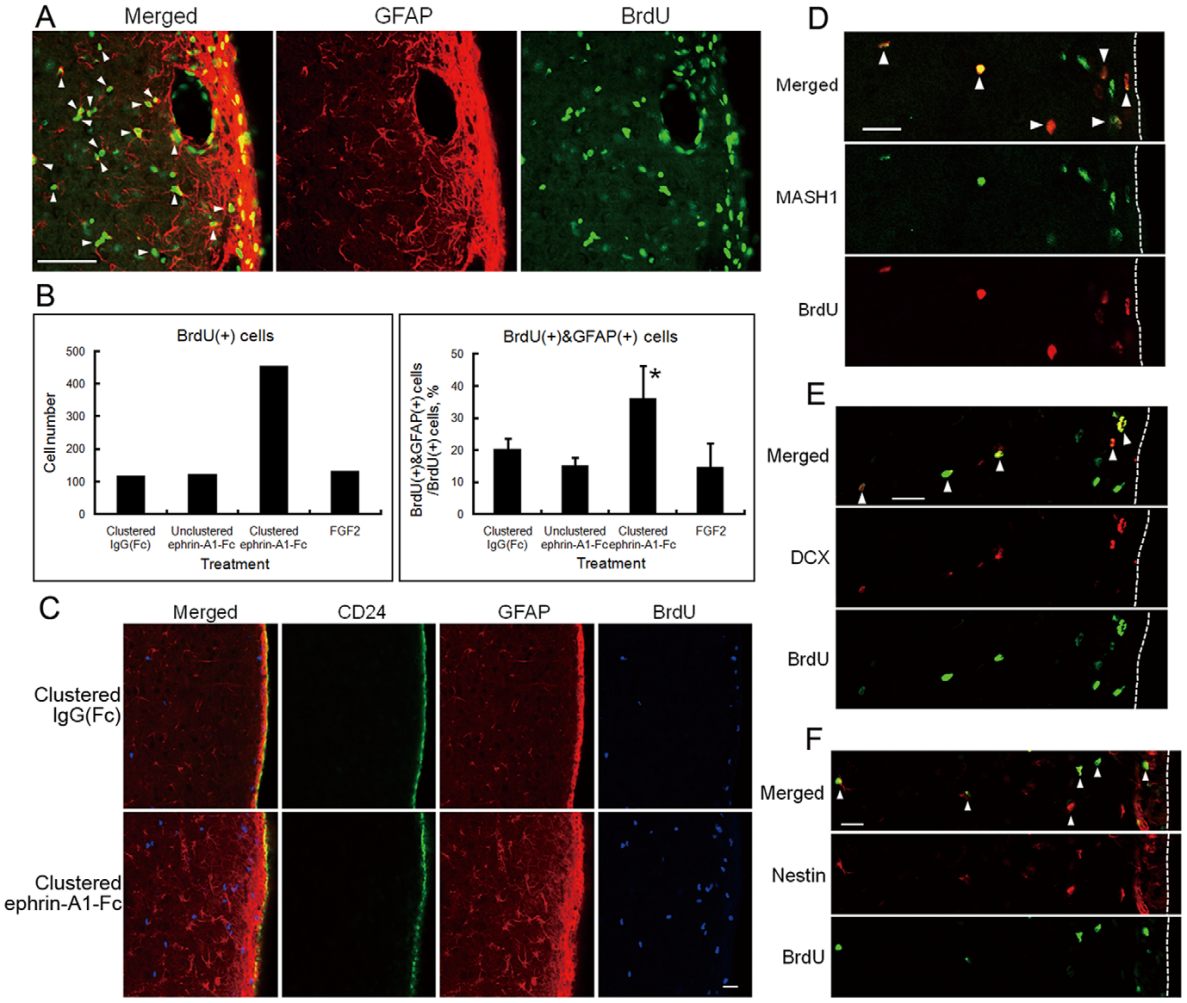
Effect of intraventricular infusion of clustered ephrin-A1-Fc on the distribution of immunostained cells in the striatum. Unilaterally lesioned rats were treated as for Fig. 5. BrdU is shown in green, and GFAP in red. (**A**) Magnification of the rectangular inset in Fig S3. White arrowheads indicate the cells positive for both BrdU and GFAP outside of the SVZ. Scale bar: 50 µm. (**B**) BrdU(+) cells and BrdU(+)&GFAP(+) cells were counted as described in the Materials and Methods. Total numbers of BrdU(+) cells in 8 animals are shown on the left, and percentages of GFAP(+) cells among BrdU(+) cells are shown on the right. Error bars represent SD. *p<0.01 (n = 8) compared to control (IgG[Fc]). (**C**) Triple staining of the lesioned side of the subventricular region after infusion of clustered ephrin-A1-Fc or IgG(Fc). CD24, green; GFAP, red; BrdU, blue. Scale bar: 20 µm. (**D**) Staining for MASH1 (green) and BrdU (red) in and around the SVZ 6 weeks after infusion of clustered ephrin-A1-Fc. White arrowheads indicate the cells positive for both MASH1 and BrdU. (**E**) Staining for Doublecortin (DCX) (red) and BrdU (green) in and around the SVZ 6 weeks after infusion of clustered ephrin-A1-Fc. White arrowheads indicate the cells positive for both DCX and BrdU. (**F**) Staining for Nestin (red) and BrdU (green) in and around the SVZ 6 weeks after infusion of clustered ephrin-A1-Fc. In (D), (E) and (F), the lateral ventricle is toward the right side of the panel, and the dotted line indicates the border between the lateral ventricle and the ependymal cell layer. Scale bar: 20 µm.

In rats that received 7-day continuous infusion of clustered ephrin-A1-Fc (3 µg/day) into the lesioned side of the lateral ventricle together with intraperitoneal 7-day BrdU injection (80 mg/kg twice a day), the infused side showed many BrdU(+) cells, densely distributed throughout the striatum and with high localization in the SVZ (Fig S3). In the ephrin-A1-Fc infused side of the brain the SVZ became thicker, and many BrdU(+) cells in the SVZ and the adjacent area were also positive for GFAP (Fig. 6A), suggesting that they are possibly adult neural stem cells capable of further neuronal differentiation. However, it cannot be excluded that GFAP (+) cells are astrocytes [2].

We counted the number of GFAP(+) cells in the striatum 500–800 µm lateral from the ventricular surface in 8 microscopic areas after treatment. Clustered ephrin-A1-Fc increased the number of BrdU(+) cells as well as the percentage of GFAP(+)BrdU(+) cells over control (IgG[Fc]) (Fig. 6B). In the same study, unclustered ephrin-A1-Fc or FGF2 did not increase the BrdU(+) or GFAP(+) cells. Microglial cells were also examined immunohistochemically by staining for ionized calcium-binding adapter molecule 1 (Iba1). These consistently represented 32% to 35% of BrdU(+) cells in response to any treatment (data not shown), suggesting that they presumably appeared in response to 6OHDA damage [39].

The same SVZ cells on the lesioned side were examined by triple-staining for GFAP, CD24, and BrdU (Fig. 6C). When clustered IgG(Fc) was infused, the lesioned side of the SVZ showed a thin layer of GFAP(+) cells beneath a monolayer of CD24–positive ependymal cells facing the lateral ventricle. However, clustered ephrin-A1-Fc induced thickening of the layer of GFAP(+) cells without affecting the monolayer of ependymal cells. We defined the width of SVZ as the thickness of GFAP staining beneath the CD24 positive ependymal cells on the striatal side, and measured the width in coronal sections at the middle of antero-posterior and dorso-ventral axes. Rats infused with clustered IgG(Fc) and ephrin-A1-Fc exhibited the width of 13.3±3.1 and 35±4.5 µm, respectively (mean ± SD; n = 6; *p*<0.001).

We also characterized the BrdU(+) cells in and around the SVZ using neural progenitor cell markers. As shown in Fig. 6D, E, and F, many BrdU(+) cells stained positive for MASH1, a marker for transit-amplifying progenitor cells [40], [41], and for Doublecortin (DCX), a marker for neuroblasts, or Nestin, a marker for neural stem cells and transit-amplifying progenitor cells. They are present not only in the SVZ but also in the adjacent striatum, supporting the migration scheme of BrdU(+) cells from the SVZ to the striatum.

### Differentiation of BrdU(+) cells to Neurons after Intraventricular Infusion of Clustered Ephrin-A1-Fc

**Figure 7:**
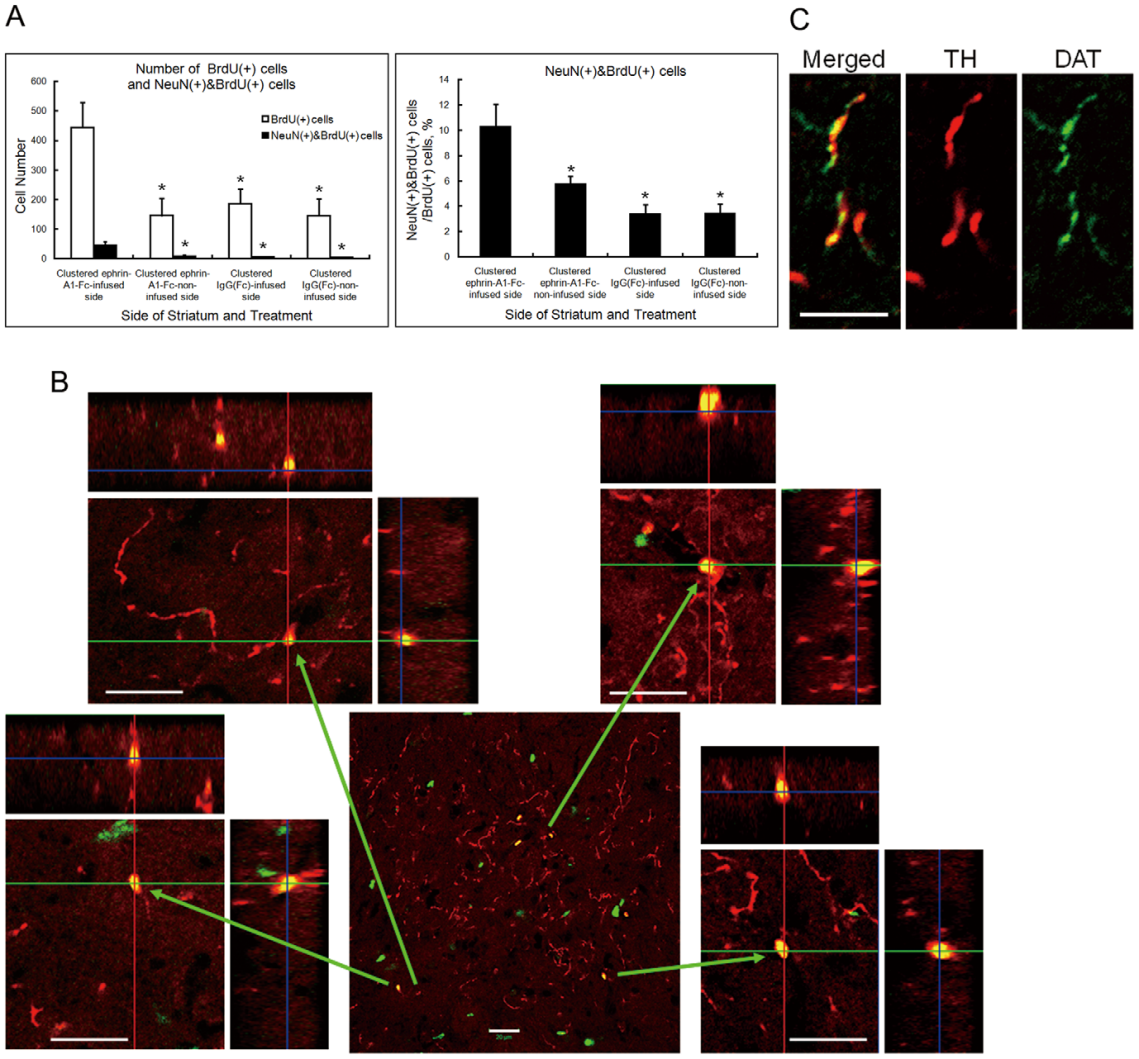
Differentiation of BrdU(+) cells to neuronal cells in the striatum. The brain of a unilaterally lesioned rat treated as in Fig. 6 was sectioned coronally and immunostained for NeuN and BrdU. (**A**) Left panel, number of cells positive for BrdU alone (white bars) and for both BrdU and NeuN (black bars) in the striatum. Cell numbers were counted in 6 animals using the dynamic cell count program of the Keyence fluorescence microscopy system. *p<0.01 (n = 6) compared to values for the side of clustered ephrin-A1-Fc infusion. Right panel, ratio of the number of NeuN(+)&BrdU(+)cells to that of BrdU(+) cells. *p<0.01 (n = 6) compared to values for the side of clustered ephrin-A1-Fc infusion. Error bars indicate SD. (**B**) BrdU(+) cells differentiating to the TH(+) neuronal lineage as detected by confocal microscopy. BrdU(+) nuclei are shown in green, and cytoplasmic TH in red. Merged regions are shown in yellow. 3D confocal photomicrographs are indicated by vertical (red) and horizontal (green) lines. Blue lines indicate the depth of the confocally sectioned plane relative to the top surface located close to the enface micrograph. Scale bar: 20 µm. (**C**) Co-localization of dopamine transporter (DAT) and TH in a regenerated neuron fiber. Sections were stained for DAT (green) and TH (red), and analyzed by confocal microscopy. Scale bar: 10 µm.

To study whether BrdU(+) cells differentiate to neurons, we counted the numbers of BrdU(+) cells and those of cells double-labeled for NeuN (neuronal nuclei) and BrdU 4 weeks after a 1-week infusion of clustered ephrin-A1-Fc in striatal sections of the unilaterally lesioned rats (Fig. 7A; Fig S4). Clustered ephrin-A1-Fc increased the number of BrdU(+) cells on the lesioned, infused side of the striatum significantly more than 2-fold compared to the lesioned, clustered IgG(Fc)-infused side (*p*<0.01; 444.8±83.5 vs. 186.8±48.9; n = 6). Clustered ephrin-A1-Fc did not affect the number of BrdU(+) cells on the contralateral side of the striatum in the same rats. The percentage of cells double-labeled for NeuN and BrdU also increased significantly on the lesioned, clustered ephrin-A1-Fc-infused side compared to the lesioned, clustered IgG(Fc)–infused side (10.30%±1.75% vs. 3.39%±0.76%; *p*<0.01; n = 6).

Importantly, some of the BrdU(+) cells were also TH(+) (Fig. 7B). These were distributed most densely in the striatum close to the anteroventral region of the clustered ephrin-A1-Fc-infused lateral ventricle. Three dimensional confocal imaging of 6 areas (9×10^4^ µm^2^/area and 10 µm thickness) of this region from 3 lesioned, clustered ephrin-A1-Fc-infused rats revealed 42 TH(+) cells among 488 BrdU(+) cells (8.6±1.14%; n = 3). No TH(+) cells were identified in the lesioned side of the striatum infused with clustered IgG(Fc), unclustered ephrin-A1-Fc, or FGF2. Almost all TH(+) cells were positive for dopamine transporter (Fig. 7C).

### Vascular Development after Intraventricular Infusion of Clustered Ephrin-A1-Fc

**Figure 8:**
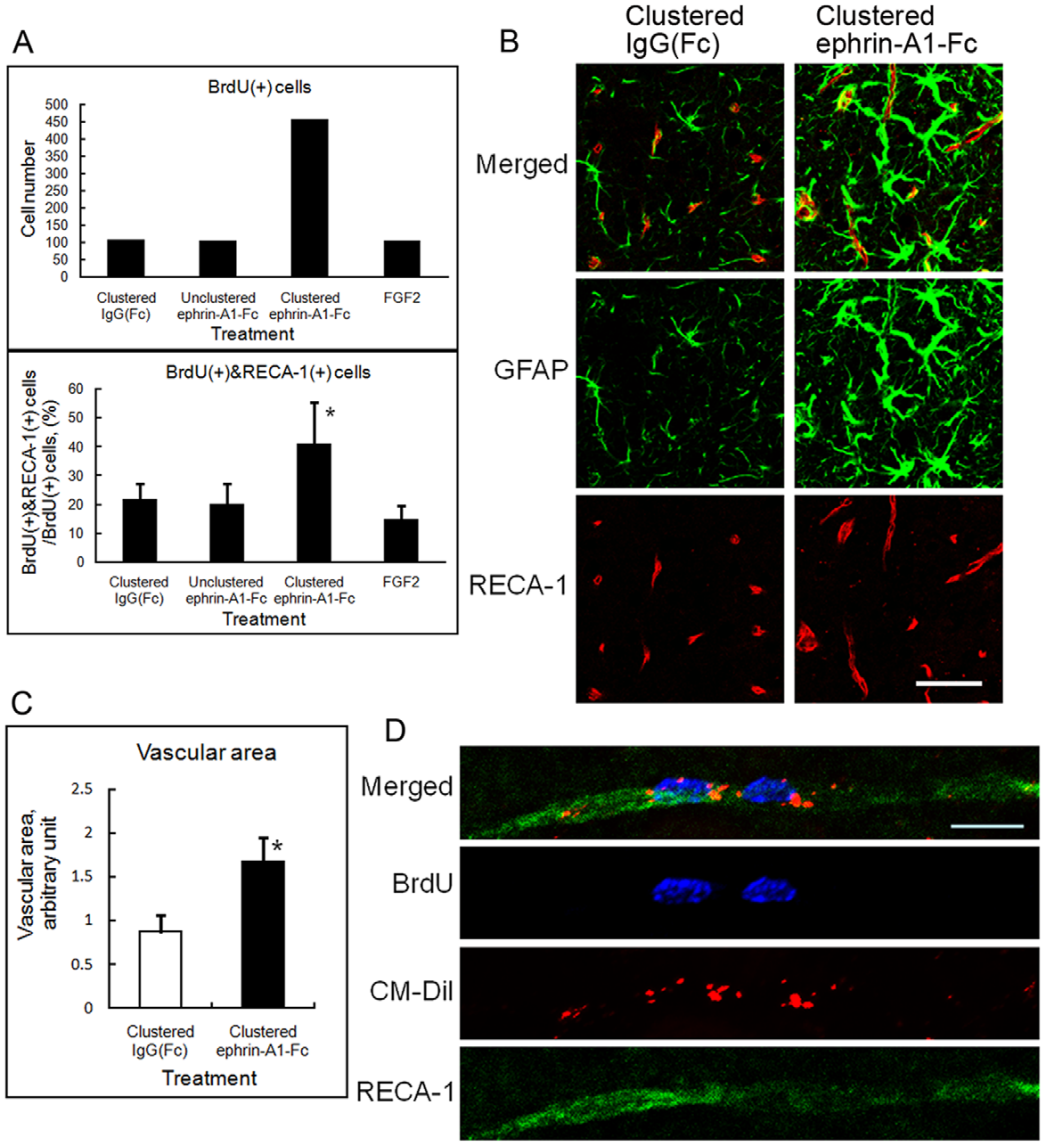
Effect of clustered ephrin-A1-Fc on vascular formation in the rat striatum. (**A**) Distribution of BrdU(+) endothelial cells. Brain of the unilaterally lesioned rats 6 weeks after infusion of clustered ephrin-A1-Fc were sectioned coronally and stained for BrdU and Rat Endothelial Cell Antigen-1 (RECA-1). Numbers of the BrdU(+) cells and BrdU(+)&RECA-1(+) cells were counted as describe in the Materials and Methods. Total numbers of BrdU(+) cells in 8 animals are shown on the top, and percentages of RECA-1(+) cells among BrdU(+) cells are shown on the bottom. Error bars represent SD. *p<0.01 (n = 8) compared to control (IgG[Fc]). (**B**) Magnified confocal micrographs of insets in Fig S5. Scale bar: 50 µm. (**C**) Quantification of endothelial cell area. Coronal sections of striatum were stained for RECA-1 as in Fig S5, and the RECA-1 stained area was quantified using an ImageJ computer program (NIH). The areas taken for measurements were as described in the Materials and Methods. The values from 6 animals were analyzed statistically. *p<0.01 (n = 6). Error bars represent SD. (**D**) Co-localization of BrdU, CM-DiI, and RECA-1 in endothelial cells of the striatum. Sections taken from the rats treated as in Fig. 5B with intraventricular CM-DiI injection were stained for BrdU and RECA-1 and subjected to 3D confocal microscopy. Micrographs are the all-in-focus compilation of 12 confocal micrographs at 0.5 µm intervals.

Ephrin-A1 induces angiogenesis in tumors as well as in embryonic and adult tissues [19]. To study whether the same effect of ephrin-A1 can be detected in the brain, clustered ephrin-A1-Fc (3 µg/day) was infused into the lesioned side of the lateral ventricle in the unilaterally lesioned rats. Brain taken from the rats 6 weeks after the start of infusion were sectioned coronally and stained for BrdU and Rat Endothelial Cell Antigen-1 (RECA-1). Treatment with clustered ephrin-A1-Fc increased BrdU(+) cells and enhanced the percentage of BrdU(+) RECA-1(+) cells (Fig. 8A). In the striatum of clustered ephrin-A1-Fc-infused rats, GFAP(+) astrocytic cells were juxtaposed to RECA-1(+) endothelial cells (Fig. 8B; Fig S5), and the endothelial cell area measured as the area of RECA-1 staining was almost twice that of clustered IgG(Fc)-injected rats (Fig. 8C). In the experiment using intraventricular injection of CM-DiI right before infusion of clustered ephrin-A1-Fc, some endothelial cells were double-labeled with CM-DiI and BrdU, suggesting that they were derived from the cells once facing the lateral ventricle, and presumably migrated to the striatum (Fig. 8D).

## Discussion

The present results indicate that subventricular cells are induced to increase their number, migrate to the striatum, and differentiate in the striatum after injection of clustered ephrin-A1-Fc into the lateral ventricle. A recent report indicates that normal neural stem cells (NSCs, B1 cells) in the SVZ possess a monociliated process that directly contacts the ventricular lumen [42]. A cluster of these apical processes is surrounded by ependymal cells. The cilia of the ependymal cells (E1 and E2) encircling the B1 stem cell processes may serve to concentrate soluble factors in the cerebrospinal fluid and induce contact of these factors with the apical processes of B1 cells. Similar signaling via primary cilia of stem cells is reported with hedgehog signals in the SGZ [43]. Alternatively, ependymal cells surrounding B1 cells might mediate signal to NSCs. However, it is less likely that clustered ephrin-A1-Fc diffuses through the ependymal layer into the brain tissue as it has a large mass, even if ependymal cells do not have tight junctions [44]. In either case, intraventricular infusion of clustered ephrin-A1-Fc appears to transform the subventricular stem cell niche, enlarging the width of the subventricular zone without affecting the ependymal layer. We have also shown that cells sensitive to clustered ephrin-A1-Fc are localized to the SVZ; the recombinant ephrin-A1-Fc affected only the ventricular side when injected into the striatum close to the ventricle. These findings strongly support that the subventricular NSPCs are the major cells affected by injection of clustered ephrin-A1-Fc into the lateral ventricle.

Recent studies show complicated results on the function of ephrins and Ephs in neurogenesis. Ephrins A2 and A5, and their receptor EphA7, regulate neural stem cell survival and proliferation in the embryonic telencephalon [23]. Ephrin-A2 reverse signaling mediates antiproliferative effects in the adult SVZ [22]. Endogenous ephrin-A2 and -A3 expressed in astrocytes appear to form an inhibitory niche that negatively regulates neural progenitor cell proliferation in adult mammalian brain areas other than the SGZ of the hippocampus and the SVZ [45]. On the other hand, EphA4 is shown to be important to maintain postnatal and adult neural stem cells in vivo [21], and EphA2, A3 and A4 appears to be important for neuronal differentiation [20]. Some ephrin-Bs and EphBs appear to be involved in suppression of NSPC proliferation in the neurogenic niche [25], [26], [28], whereas others are involved in proliferation or at least maintenance of NSPCs [24], [27], [29]. Of course, different ephrins might have different functions in vivo. However, at least a part of these complicating effects could be explained by the experimental system used for each study. We suspect that our strategy of ectopic application of clustered ephrin-A1-Fc from inside the ventricle, in which clustered ephrin stays out of the tissue and affects only from the side of cerebral fluid, might differ from endogenous expression or deletion via genetic engineering in that it elicits forward signaling with barely affecting the reverse signaling. However, we cannot completely exclude the possibility that some clustered ephrin diffuses into the SVZ tissue, and stimulates the forward signaling and blocks the reverse signaling.

Pulse-chase experiments and CM-DiI labeling from the ventricular side demonstrated that BrdU(+) cells migrated extensively from the SVZ to the striatum after injection of clustered ephrin-A1-Fc into the lesioned side of the lateral ventricle. Staining of the cells with several cell markers, Nestin [46], MASH1 [40], and DCX [47], also support that the migrating cells in and around the SVZ are neural stem cells, transit-amplifying cells and neuroblasts. Migration occurred on the lesioned side of the striatum only after ipsilateral intraventricular injection. The normal side was barely affected unless ephrin was injected into this side of the lateral ventricle. In addition to the above-mentioned signaling mechanisms via stem cell cilia or ependymal cells [42], increased sensitivity of cells residing on the damaged side might play a role in this effect [8], [9]. Migration of BrdU(+) cells via the default rostral migratory stream, as shown in our control studies, was also augmented heavily by clustered ephrin-A1-Fc and moderately by FGF2, suggesting that the effect of clustered ephrin-A1-Fc on striatal migration is not a nonspecific phenomenon. The molecular mechanisms of cellular migration involve cytoskeletal changes induced by a dynamic structural shift of actin microfilaments and microtubules in response to external signals [48]. We suspect that reorganization of microfilaments mediated by RhoA activation following complex formation of FGFR, EphA4 and ephexin1, which depends on EphA4 activation, is at least partially involved in the mechanism of stem cell migration into the striatum [13], [36]. In this case, FGFR is responsible for activation of the Rho family guanine nucleotide exchange factor, ephexin1. We have shown that all four classes of FGFRs and ephexin1 are also expressed in the SVZ, and EphA4, a predominant Eph that binds to recombinant ephrin-A1-Fc in the SVZ, is phosphorylated in the SVZ cells of rats intraventricularly infused with clustered ephrin-A1-Fc. Examining this way of signaling, we performed injection of SU5402, an FGFR inhibitor, into the lateral ventricle to test if the function of clustered ephrin-A1-Fc can be blocked. We have preliminary data to be completed showing that the inhibitor suppresses not only migration of BrdU(+) cells into the striatum but also recovery from the typical rotational behavior induced by apomorphine injection in the rats with a unilaterally lesioned nigrostriatal dopaminergic pathway.

Injection of clustered ephrin-A1-Fc into the lateral ventricle induced histologic and behavioral improvements in the unilaterally lesioned rats. A single injection (3 µg) into the lesioned side of the lateral ventricle improved apomorphine-induced rotating behavior significantly. Continuous infusion for 1 week with a dose of 2 µg/day produced even greater improvements. The effect of ephrin injection on these histologic and behavioral improvements was evident 12 weeks after ephrin injection. This behavioral recovery is most likely to be caused by differentiation of NSPCs to astrocytes and dopaminergic neurons. Regeneration of damaged but surviving residual dopaminergic fibers might be induced by infusion of clustered ephrin-A1-Fc into the lateral ventricle, and had some functions in the behavioral improvement. However, we waited for 6 weeks after 6OHDA injection, and confirmed almost complete depletion of dopaminergic neural fibers in the striatum right before ephrin-A1-Fc infusion. We found that a major proportion of BrdU(+) cells migrating through the striatum differentiated to astrocytes when stimulated by clustered ephrin-A1-Fc from inside the lateral ventricle, and that differentiation of BrdU(+) cells to dopaminergic neurons also occurred in the striatum. Differentiation of subventricular NSPCs to dopaminergic neurons by ephrin-A ligands has been reported in an *in vitro* study, and appears to be mediated by MAP kinase activation [20]. Astrocytes generally interact with neurons directly [49] and play an important role in supporting neurons. In the RMS, chains of migrating adult neuroblasts are ensheathed by astrocytes [50]. They express cytokines and cytokine receptors; other factors that act as neuroprotective agents of damaged neurons [51], [52]; constituents of the niche microenvironment [53]; differentiation/maturation factors [54]; and synaptogenic factors [54]. Thus, newly differentiated astrocytes in response to clustered ephrin-A1-Fc injection are suspected to play a critical role in differentiation of neural stem cells to dopaminergic neurons and their maintenance in the striatum.

Another finding is that clustered ephrin-A1-Fc increased angiogenesis in the striatum on the injected side. Ephrins and their Eph receptors are known to regulate angiogenesis [19]. Originally, B-class receptors and ligands were considered important players in endothelial cell migration and differentiation, leading, in concert with other growth factors, to capillary formation. Recent studies demonstrate the additional involvement of ephrin-A1 and EphA2 in postnatal and tumor angiogenesis [55]. Intraventricularly injected clustered ephrin-A1-Fc likely increased capillary formation via binding to receptors on ependymal cells or B1 neural stem cells that directly contact the ventricular fluid, as subventricular capillary vessels locate far from the ventricular surface [7], [42]. In either case, signaling for endothelial cell proliferation appears to be indirect. However, the finding of endothelial cells double-labeled with BrdU and CM-DiI suggests a possibility that neural stem cells differentiate to endothelial cells. This kind of transdifferentiation has been reported in neural stem cells in vivo [56]. Subventricular capillary vessels, once formed, are permeable to small molecules; this facilitates access of stem cells and transit-amplifying cells to molecules in the bloodstream such as growth factors, hormones, and nutrients [7]. Ephrin-A1 further increases this vascular permeability upon stimulation of its receptor EphAs as demonstrated in the lung [57]. Thus, clustered ephrin-A1-Fc appears to stimulate angiogenesis by inducing stem cell proliferation in the SVZ. In turn, the enhanced angiogenesis would help increase the stem cell proliferation as well.

In conclusion, injection of clustered ephrin-A1-Fc into the lateral ventricle induced transformation of the subventricular niche, resulting in increase of BrdU(+) NSPCs in and around the SVZ, their migration to the striatum, their differentiation to astrocytes and neuronal cells, including dopaminergic cells, and angiogenesis. Owing to this dopaminergic regeneration supported by increasing numbers of astrocytes and capillary vessels in the striatum, behavioral abnormalities caused by lesion to the nigrostriatal pathway decreased dramatically. These findings may lead to the development of new therapeutic approaches for neurodegenerative diseases such as Parkinson's disease and related disorders in humans.

## Supporting Information

Figure S1 **Effect of clustered ephrin-A1-Fc injection in rats with chemical lesions in the unilateral nigrostriatal dopaminergic pathway.** (**A and B**) Effect of intraventricular injection of clustered ephrin-A1-Fc on the regeneration of TH–positive cells. Six weeks after clustered ephrin-A1-Fc or IgG(Fc) was injected into the ventricle ipsilateral to the lesioned side, coronal sections of the striatum near the lateral ventricle were stained for TH (red in A and green in B). (**A**) Lesioned (left panel) and normal (right panel) sides of the subventricular and striatal area after injection of clustered ephrin-A1-Fc. (**B**) Lesioned side after injection of clustered IgG(Fc) (left panel) or clustered ephrin-A1-Fc (right panel). Scale bar: 100 µm.
(DOC)

Figure S2 **Effect of intraventricular infusion of ephrin-A1-Fc on the distribution of BrdU(+) cells in the striatum and olfactory bulb.** Unilaterally lesioned rats were infused with clustered IgG(Fc) (3 µg/day), unclustered ephrin-A1-Fc (3 µg/day), clustered ephrin-A1-Fc (3 µg/day), or FGF2 (50 ng/day) into the lateral ventricle of the lesioned side with simultaneous intraperitoneal injection of BrdU twice a day. Brains were removed 6 weeks after the start of infusion, sliced into 40-µm thick coronal sections, and stained for BrdU. Numbers of BrdU(+) cells were counted in 8 areas of coronal sections of striatum or sagittal sections of the granule cell layer of the olfactory bulb as described in Materials and Methods. We used 8 animals. Error bars represent SD. *p<0.01 (n = 8) compared to clustered IgG(Fc).
(DOC)

Figure S3 **Effect of intraventricular infusion of clustered ephrin-A1-Fc on the distribution of BrdU(+) and GFAP(+) astrocytic cells.** Unilaterally lesioned rats were treated as for Fig. 5. Confocal micrographs are shown with BrdU in green and GFAP in red. The rectangular inset is magnified in Fig. 6A with split colors. Scale bar: 100 µm.
(DOC)

Figure S4 **Differentiation of BrdU(+) cells to neuronal cells.** The brain of a unilaterally lesioned rat treated as in Figs. 5 and 6 was sectioned coronally and immunostained for NeuN and BrdU. Immunostained cells in the striatum were detected by confocal microscopy. Cells positive for both NeuN and BrdU are indicated by arrowheads. Scale bar: 20 µm.
(DOC)

Figure S5 **Effect of clustered ephrin-A1-Fc on vascular formation in the rat striatum.** Clustered ephrin-A1-Fc was injected into the lesioned side of the lateral ventricle in the unilaterally lesioned rats. Brains taken 6 weeks after injection were sectioned coronally and stained for GFAP (green) and RECA-1 (red) and with DAPI (nuclei; blue). The rectangular insets are shown in Fig. 8B. Scale bar: 100 µm.
(DOC)

Table S1 **Oligonucleotide primers used in this Study.** Prefix r indicates rat; suffixes -F and -R indicate forward and reverse, respectively. *Gapdh*, glyceraldehyde 3 phosphate dehydrogenase; *Frs2α*, fibroblast growth factor receptor substrate 2α; *Fgfr*, fibroblast growth factor receptor.
(DOC)

Table S2 **Antibodies used in this Study.** FRS2α, fibroblast growth factor receptor substrate 2α; FGFR, fibroblast growth factor receptor.
(DOC)
