# Supplementary material for: Ephrin-A1-Mediated Dopaminergic Neurogenesis and Angiogenesis in a Rat Model of Parkinson's Disease
Source: PLoS One. 2012 Feb 20;7(2):e32019. doi: 10.1371/journal.pone.0032019 (PMC3282790; doi:10.1371/journal.pone.0032019)
Supplement: Table S1 — Oligonucleotide primers used in this Study. Prefix r indicates rat; suffixes -F and -R indicate forward and reverse, respectively. Gapdh, glyceraldehyde 3 phosphate dehydrogenase; Frs2α, fibroblast growth factor receptor substrate 2α; Fgfr, fibroblast growth factor receptor. (DOC) [file pone.0032019.s006.doc]

**Table S1**

| Primer | Sequence (5'–3') | Length (bp) | NCBI code |
| --- | --- | --- | --- |
| r*Epha1*-F | ACCAGGATGAAGAATGGCAC | 580 | NM_001107858 |
| r*Epha1*-R | AACTGGCCCATGATAGTTGC |
| r*Epha2*-F | AAAGCCGGCTACACTGAGAA | 411 | NM_001108977 |
| r*Epha2*-R | GGCTGTCCATCGAATAGGAA |
| r*Epha3*-F | TACGGAGAAAGGCCGTATTG | 351 | NM_031564 |
| r*Epha3*-R | CTCCACACCCGTGAAGATTT |
| r*Epha4*-F | TCGAGGCTCCTGTGTCAACAACTC | 642 | NM007936 |
| r*Epha4*-R | GATGATGGTGCTGCTTGGTTG |
| r*Epha5*-F | CAAGCCTAATATGGCGGTGT | 627 | NM_001169137 |
| r*Eph*a*5*-R | CCCTGGAAAGTCCAAAGTCA |
| r*Epha6*-F | GCAAAGTCTCTGATTTCGGC | 425 | XM_001057372 |
| r*Epha6*-R | GGATTCTGGCATTACGAGGA |
| r*Epha7*-F | GGAAAAATTCCAGTAAGGTG | 518 | NM_134331 |
| r*Epha7*-R | ATCCCTAAACTCATCACATC |
| r*Epha8*-F | GGCTATAGCAAGGCATTCCA | 392 | XM_342952 |
| r*Epha8*-R | AGGCGGATGATGTTAGGATG |
| r*Epha10*-F | CCTGTGATGGCAAAGGAAGT | 523 | NM_001135709 |
| r*Epha10*-R | ACATAGCCCATTTCCGACAG |
| r*Gapdh*-F | ATGGGAAGCTGGTCATCAAC | 440 | AF106860 |
| r*Gapdh*-R | GGATGCAGGGATGATGTTCT |
| r*Efna1*-F | CCGCTGCTGACCGCCACATC | 358 | NM_053599 |
| r*Efna1*-R | TGGTAGATAGGTTTGGAGAT |
| r*Efna2*-F | TGGAGGTCAGCATCAATGAC | 351 | NM_001168670 |
| r*Efna2*-R | ACAGGGTCTCATTGGTTGGA |
| r*Efna3*-F | ATGCGGTATACTGGAACAGC | 246 | XM_574979 |
| r*Efna3*-R | AGAACTTGATGGGGCTGTGC |
| r*Efna4*-F | TTGCCCCTGCTGCGGACTGT | 462 | NM_001107692 |
| r*Efna4*-R | CTTGCAGCAGACAGACACCT |
| r*Efna5*-F | CTGGCTAGGCGTGATGTTGC | 480 | NM_053903 |
| r*Efna5*-R | ACTTTGAGCTTTAGGCAGGA |
| r*Ephexin1*-F | GAGGCCATGTTTGAGTTGGT | 422 | NM_001136241 |
| r*Ephexin1*-R | ATCCTCTGGAAGGGCAAGAT |
| r*Frs2α*-F | CTGGCTATGACAGCGATGAA | 594 | NM_001108097 |
| r*Frs2α*-R | GCAAGTTGGACATAGCAGCA |
| r*Fgfr1*-F | AGG CAA TCT TCG GGA GTA TCT GC | 561 | NM_024146 |
| r*Fgfr1*-R | TCT TCC ACC AAC TGC TTG AAA GT |
| r*Fgfr2*-F | TTTATAGTGATGCCCAGCCC | 590 | NM_012712 |
| r*Fgfr2*-R | TTCCCACTTTGGATCCTCTG |
| r*Fgfr3*-F | AGAACAAGTTTGGCAGCATC | 374 | NM_053429 |
| r*Fgfr3*-R | CACCAGCCACGCAGAGTGAT |
| r*Fgfr4*-F | TCGATCCACTTTGGGAGTTC | 523 | NM_001109904 |
| rFgfr4-R | TCAGCGATCTTCATCACGTC |
