# Supplementary material for: Ephrin-A1-Mediated Dopaminergic Neurogenesis and Angiogenesis in a Rat Model of Parkinson's Disease
Source: PLoS One. 2012 Feb 20;7(2):e32019. doi: 10.1371/journal.pone.0032019 (PMC3282790; doi:10.1371/journal.pone.0032019)
Supplement: Table S2 — Antibodies used in this Study. FRS2α, fibroblast growth factor receptor substrate 2α; FGFR, fibroblast growth factor receptor. (DOC) [file pone.0032019.s007.doc]

**Table S**2

| Antigen | Antibody | Source | Code number | Dilution |
| --- | --- | --- | --- | --- |
| MASH1 | Mouse monoclonal | Johnson, J.E.  UT Southwestern Medical Center |  | 1/100 |
| Nestin | Mouse monoclonal | Chemicon International | rat-401 | 1/500 |
| Nestin | Rabbit polyclonal | Santa Cruz | sc-20978 | 1/500 |
| Neuronal class III beta-tubulin | Mouse monoclonal | Covance | MMS-435P; Clone TUJ1 | 1/500 |
| Glial fibrillary acidic protein (GFAP) | Mouse monoclonal | Sigma-Aldrich | G3893 | 1/1000 |
| Glial fibrillary acidic protein (GFAP) | Rabbit polyclonal | DAKO | Z0334 | 1/1000 |
| Doublecortin | Goat polyclonal | Cell Signaling | 4604 | 1/200 |
| NeuN  (neuronal nuclei) | Mouse monoclonal | Chemicon International | MAB377 | 1/500 |
| Tyrosine hydroxylase (TH) | Rabbit polyclonal | Chemicon International | AB152 | 1/500 |
| Tyrosine hydroxylase (TH) | Mouse monoclonal | Chemicon International | MAB318 | 1/500 |
| Dopamine transporter (DAT) | Rat monoclonal | Chemicon International | MAB369 | 1/500 |
| EphA1 | Rabbit polyclonal | Santa Cruz | sc-925 | 1/200 |
| EphA2 | Rabbit polyclonal | Santa Cruz | sc-924 | 1/200 |

| Antigen | Antibody | Source | Code number | Dilution |
| --- | --- | --- | --- | --- |
| EphA3 | Rabbit polyclonal | Santa Cruz | sc-920 | 1/200 |
| EphA4 | Rabbit polyclonal | Santa Cruz | sc-921 | 1/200 |
| EphA5 | Rabbit polyclonal | Santa Cruz | sc-1014 | 1/200 |
| EphA6 | Goat polyclonal | Santa Cruz | sc-8172 | 1/200 |
| EphA7 | Rabbit polyclonal | Santa Cruz | sc-1015 | 1/200 |
| EphA8 | Goat polyclonal | Santa Cruz | sc-7287 | 1/200 |
| Rat endothelial cell antigen-1 (RECA-1) | Mouse monoclonal | Monosan | MON6005; Clone HIS-52 | 1/80 |
| Ionized calcium-binding adapter molecule 1 (Iba1) | Rabbit polyclonal | Wako Pure Chemical Industries | 019-19741 | 1/500 |
